# OBAMA: OBAMA for Bayesian amino-acid model averaging

**DOI:** 10.7717/peerj.9460

**Published:** 2020-08-04

**Authors:** Remco R. Bouckaert

**Affiliations:** 1 School of Computer Science, University of Auckland, Auckland, New Zealand; 2 Max Planck Institute for the Science of Human History, Jena, Germany

**Keywords:** Amino acid model, Protein model, Phylogenetics, Bayesian analysis, Statistical phylogenetics, Substitution model, Site model, Bayesian model averaging, BEAST, Gamma rate heterogeneity

## Abstract

**Background:**

Bayesian analyses offer many benefits for phylogenetic, and have been popular for analysis of amino acid alignments. It is necessary to specify a substitution and site model for such analyses, and often an ad hoc, or likelihood based method is employed for choosing these models that are typically of no interest to the analysis overall.

**Methods:**

We present a method called OBAMA that averages over substitution models and site models, thus letting the data inform model choices and taking model uncertainty into account. It uses trans-dimensional Markov Chain Monte Carlo (MCMC) proposals to switch between various empirical substitution models for amino acids such as Dayhoff, WAG, and JTT. Furthermore, it switches base frequencies from these substitution models or use base frequencies estimated based on the alignment. Finally, it switches between using gamma rate heterogeneity or not, and between using a proportion of invariable sites or not.

**Results:**

We show that the model performs well in a simulation study. By using appropriate priors, we demonstrate both proportion of invariable sites and the shape parameter for gamma rate heterogeneity can be estimated. The OBAMA method allows taking in account model uncertainty, thus reducing bias in phylogenetic estimates. The method is implemented in the OBAMA package in BEAST 2, which is open source licensed under LGPL and allows joint tree inference under a wide range of models.

## Introduction

To perform a Bayesian phylogenetic analysis with amino acid alignments one needs to define a site model. A site model consists of a substitution model defining the relative rates of different classes of substitutions, a frequency model defining base frequencies for the amino acids, whether there is rate heterogeneity across sites (often specified as gamma distribution (Yang, 1994)) and whether a proportion of sites is invariable (Gu, Fu & Li, 1995; Waddell & Penny, 1996). The site model is usually chosen ad hoc, or based on a maximum likelihood analysis like ProtTest (Abascal, Zardoya & Posada, 2005; Darriba et al., 2011), ModelFinder (Kalyaanamoorthy et al., 2017) or ModelTest-NG (Darriba et al., 2020). As a consequence, model uncertainty is not taken into account in the analysis, potentially leading to biased estimates, especially with smaller amounts of data.

For nucleotide alignments, a similar situation exists where it is popular to use ModelTest/jModelTest (Posada & Crandall, 1998; Posada, 2008; Darriba et al., 2012) to decide the substitution and site model. There are a number of methods allowing averaging over nucleotide substitution models in a Bayesian setting (Bouckaert, Alvarado-Mora & Rebello Pinho, 2013; Huelsenbeck, Larget & Alfaro, 2004; Wu, Suchard & Drummond, 2013) and the bModelTest method (Bouckaert & Drummond, 2017) allows averaging site models. For amino acid data, no such method has been developed so far.

Here, we present a new method that averages over site models where the substitution model is one of the available empirical models. Furthermore, it allows switching between base frequencies as specified by the empirical models listed in Table 1 and frequency estimates based on the alignment. Like bModelTest, it allows averaging over having homogenous rates for all sites or gamma distributed rate heterogeneity over sites (Yang, 1994) as well as averaging over having a proportion of invariable sites or not. The method is called **O**BAMA for **B**ayesian **A**minoacid **M**odel **A**veraging. If the phylogeny is the object of interest in the analysis and the site model can be considered a nuisance parameter, the OBAMA method handles site model uncertainty by averaging over all available models. However, site model parameters estimates are produced from an analysis with this method as well.

**Table 1:** Empirical substitution models used in OBAMA.

Model name	Reference
Blosum62	Henikoff & Henikoff (1992)
CpREV	Adachi et al. (2000)
Dayhoff	Dayhoff, Schwarz & Orcutt (1978)
DCMut	Kosiol & Goldman (2005)
FLU	Dang et al. (2010)
HIVb	Nickle et al. (2007)
HIVw	Nickle et al. (2007)
JTT	Jones, Taylor & Thornton (1992)
LG	Le & Gascuel (2008)
MtArt	Abascal, Posada & Zardoya (2007)
MtMam	Cao et al. (1998)
mtREV	Adachi & Hasegawa (1996)
RtREV	Dimmic et al. (2002)
VT	Müller & Vingron (2000)
WAG	Whelan & Goldman (2001)

The method is implemented in the OBAMA package of BEAST 2 (Bouckaert et al., 2014; Bouckaert et al., 2019) with GUI support for BEAUti making it easy to use with multiple partitions. BEAST 2 offers a range of methods in conjunction with tree estimates that can be used in combination with OBAMA, such as phylogeographical reconstructions, inference using morphological characters, and a range of distributions describing tree generating processes such as (structured) coalescent and (fossilised) birth/death processes. It is open source and available under LGPL licence. Source code, installation instructions, guides for trouble shooting and documentation can be found at https://github.com/rbouckaert/obama.

## Methods

### Site model

We consider phylogenetic site models consisting of four parts:

• a symmetric rate matrix *Q* of size 20 × 20 specifying the rate of substitution among amino acids. During the MCMC, we average over the 15 empirical substitution models listed in Table 1, using a model indicator *I*_*M*_ that identifies the model by an integer number.
• a frequency model *F* specifying the base frequencies of the 20 amino acids. Empirical models come with empirical frequencies, but we can also estimate them using MCMC.
• a model of rate variation over sites. We consider no variation at all (homogenous rates) and gamma distributed heterogeneous rates using 4 categories (Yang, 1994) with gamma shape parameter *α* estimated.
• the choice whether to include a category of invariable sites and estimate the proportion of invariable sites *p*_*inv*_ or not.

The rate matrix *Q* and frequency model *F* together specify a rate matrix *R* = *Q* × *F* that determine a transition probability matrix *P* = *e*^*Rt*^, which can be used to calculate the likelihood of a tree given the alignment efficiently using Felsenstein’s pruning algorithm (Felsenstein, 1981).

### Prior

**Figure 1:**
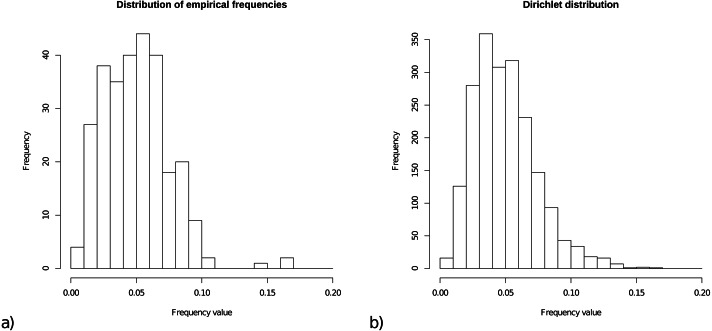
(A) Distribution of empirical frequencies for models in Table 1. (B) Dirichlet(4,4, …,4) distribution used as frequency prior which is the default for OBAMA that can be changed by the user.

For the prior on frequencies, we collected all frequencies from the 15 empirical models, and Fig. 1A shows a histogram of these 15 × 20 = 300 frequencies. The range of frequencies is from 0.006 to 0.169 with mean 0.050001. A Dirichlet prior is appropriate for a set of variables that is constrained to sum to 1. Figure 1B shows a sample of 2000 cases from a Dirichlet(4,4, …,4), which has range for samples from 0.005 to 0.167 with mean 1/20=0.05, and this statistic resembles the empirical distribution for frequencies.

**Figure 2:**
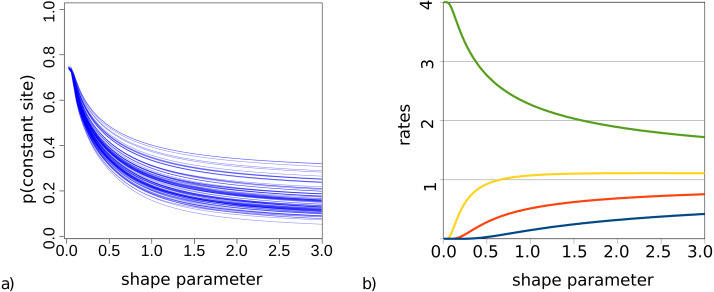
(A) Probability of a site being invariable under gamma rate heterogeneity models for the 100 trees used in the simulation study. On the *x*-axis the value of the shape parameter *α* for the gamma distribution, and on the *y*-axis the probability that an invariable site is observed under an empirical model with gamma rate heterogeneity. Lower shape parameter values lead to higher proportion of invariable sites. (B) Rate values for the four categories are a function of the shape parameter.

For bModelTest (Bouckaert & Drummond, 2017), we observed some lack of identifiability when both gamma rate heterogeneity and invariable sites were included in the true site model used to simluate data. This was earlier observed for maximum likelihood estimates of these parameters (p. 120, Yang (2014)). This phenomenon is particularly problematic when the shape parameter *α* governing gamma rate heterogeneity is small and a large number of invariable sites can be expected. To demonstrate this, Fig. 2 shows the probability of observing an invariable site for the set of trees (from our simulation study described in detail later) and substitution models with gamma rate heterogeneity using 4 categories, but no invariable sites. This is calculated using Felsenstein’s algorithm as the probability that each of the tips in a tree have exactly the same amino acid, summed over all 20 amino acids.

When we look at how rates behave with small *α* and 4 categories (Fig. 2), we notice that when *α* < 0.2 the slowest category has a rate less than 0.00015. This implies for a branch of length 1 (i.e., where the expected number of substitutions per site is 1) that for every 100 sites we expect more than 99 sites to be invariable under such rate. When *α* < 0.1 the second slowest rate decreases to 0.007 and now for the second rate we expect over 99 out of 100 sites to be invariable as well, while the remaining rate heterogeneity must be captured by the two fastest rates. At *α* < 0.05, the third rate decreases below 0.005, and almost all rate heterogeneity has to be captured by the remaining rate, which with decreasing *α* becomes 4 − *ϵ* with *ϵ* < 0.001. In this situation, the probability of invariable sites becomes 0.75 in the case of 4 categories.

This does not seem to be reasonable behaviour for this model, so our prior on *α* should exclude *α* below 0.1. Empirical results (Table 2.2 in Yang (2014)) appear to fall in a range above 0.1. Even at *α* = 0.1 there is still a high probability of invariable sites, so the identifiability problem is not completely solved, but at least somewhat diminished. Since we would like gamma rate heterogeneity only to be included in the model if there is a reasonable amount of heterogeneity in the data (which coincides with low values of *α*), by default we use an exponential prior with rate 1, truncated to exclude values below 0.1.

For the other items we chose uninformative priors. In summary, the prior consists of the product of the following independent priors:

• a uniform prior on empirical models through a uniform prior on the model indicator *I*_*M*_.
• a uniform prior on using empirical frequencies (from the empirical model indicated by *I*_*M*_) or estimated frequencies.
• a uniform prior on including gamma rate heterogeneity or not.
• a uniform prior on including a proportion of invariable sites or not.
• a Beta(1,4) prior on the proportion of invariable sites. This skews the proportion of invariable sites rather to lower than to higher proportions of invariable sites.
• a Dirichlet(4,4,…,4) prior on estimated frequencies.
• an exponential prior on the shape parameter for gamma rate heterogeneity with rate 1 and lower bound 0.1.

### MCMC proposals

For switching between substitution models, we use a uniform proposal for the model indicator *I*_*M*_, which uniformly selects an indicator value in the range of 1 to the number of empirical subsitution models included in the analysis.

For gamma rate heterogeneity and proportion of invariable sites we use the same birth and death operators as for bModelTest. For gamma rate heterogeneity, the birth and death proposal sets or unsets the category count flag and samples a new value for shape parameter *α* from the prior (by default an exponential with lower bound 0.1) when the flag is set. The proposal ratio is *d*(*α*′) for birth and 1∕*d*(*α*) for death where *d*(.) is the density used to sample from. Likewise, for setting the proportion of invariable sites flag and sampling *p*_*inv*_ from the prior (by default a Beta(1,4)). The Jacobian determinant is 1 for these proposals, so have no impact on the Hastings ratio. For moving *α*, we use the standard scale operator in BEAST 2 (Bouckaert et al., 2014), adapted so it only samples if the category count flag is set for *α*. In the same way, for *p*_*inv*_ such a scale operator is used only if the proportion of invariable sites flag is set.

The estimation of frequencies is done through Bayesian stochastic variable selection (Kuo & Mallick, 1998; Lemey et al., 2009), where we use a bit flip operator on a boolean parameter to switch between empirical model frequencies and estimated frequencies.

To estimate frequencies, efficiency of operators for sampling was conducted by comparing two operators: the delta exchange operator and the adaptive variance multivariate normal (AVMN) operator (Baele et al., 2017). A delta exchange operator randomly selects two frequencies *f*_*a*_ and *f*_*b*_, then randomly selects a value *δ* and proposes new frequencies ${f}_{a}^{{^{\prime}}}={f}_{a}+\delta $, ${f}_{b}^{{^{\prime}}}={f}_{b}-\delta $ under the condition that $0\leq {f}_{a}^{{^{\prime}}}\leq 1$ and $0\leq {f}_{b}^{{^{\prime}}}\leq 1$. An AVMN operator parameterises the space spanned by frequencies as a log-constrained sum transformed multivariate parameter and approximates the distribution of that transformed parameter using a multivariate normal distribution *N*(*M*, Σ) for which the mean vector *M* and covariance matrix Σ are learned during the MCMC run (see Baele et al. (2017) for more details). We use the settings recommended in Baele et al. (2017) for the AVMN operators in our experiments.

## Results

### Validation of model implementation

We performed a well calibrated simulation study to verify correctness of the implementation and investigated the behaviour of OBAMA on a published alignment.

#### Simulation study

For the simulation study, we simulated 100 cases under the OBAMA model, with a Yule prior parameterised by a birth rate parameter *λ*, and an uncorrelated relaxed clock (Drummond et al., 2006) using 16 taxa. We used a narrow prior on *λ* (log normal with mean 5.5 in real space and standard deviation of 0.048) in order to simulate trees with mean height of 0.44 substitutions per site with a range from 0.17 to 1.0. Broader priors would lead to larger trees which can make it impossible to infer trees due to saturation, even if extremely large sequence lengths were used. For the standard deviation of the relaxed clock, we used a gamma (with shape = 0.5 and scale =0.4) distribution, which has mean 0.2 giving moderate rate variation for most cases.

We obtained 100 instances of trees and parameter values by sampling from the prior using MCMC. By using a sufficiently long chain, samples were guaranteed to be independent, which was confirmed by inspecting the trace log in Tracer (Rambaut et al., 2018). For each of these cases, we simulated data under the model parameters distinguishing 8 cases: any combination of with/without estimated frequencies, with/without gamma rate heterogeneity and with/without a proportion of invariable sites, providing a total of 800 alignments over 16 taxa with 200 amino acids. For each of these 800 alignments, an MCMC analysis was done under the OBAMA site model, Yule tree prior and uncorrelated relaxed clock model, all with the same priors and hyper priors as used to sample the data. Data files used in simulation study can be found at https://github.com/rbouckaert/obama/releases/tag/data.

Table 2 summarises the results. The first four rows in the table represent the number of analyses where the true substitution model is in the 95% credible set, so the expected number is 95. All following rows represent the number of runs in which the true value was in the estimated 95% HPD interval. Entries with an ‘x’ represent cases where the true model does not use the associated parameters, making it impossible to define coverage. For an experiment with 100 instances, coverage is distributed according to a binomial distribution with *p* = 0.95 and *N* = 100, so the expected coverage is 95 with a 95% HPD of 90 to 99, and we expect 95 out of 100 entries in the table to be in the range 90-99. Note that some of the frequencies have a slightly lower coverage, but not less than could be expected with this many entries. Overall, the table shows that models could be recovered from data simulated under such model, and it provides some confidence that our implementation is correct.

**Table 2:** Substitution model coverage in percentage for simulation study for 16 taxa and 250 sites with an expected value for each entry of 95. All sequences were simulated under an uncorrelated relaxed clock. Columns represent models under which the data was simulated: +G indicates that gamma rate heterogeneity was used, +I indicates a proportion of invariable sites was used, +F indicates frequencies were drawn from a Dirichlet(4, …,4) distribution instead of using empirical model frequencies. Overall, the table shows that models could be recovered from data simulated under such model.

True model:	–	+G	+I	+G+I	+F	+G+F	+I+F	+G+I+F
Model Indicator	100	100	100	100	100	100	100	100
has Invariable Sites	100	100	99	100	100	100	100	100
has Gamma Rates	100	100	100	100	100	100	99	100
use External Freqs	100	100	100	100	100	100	100	100
gamma Shape	x	99	x	92	x	97	x	90
Proportion Invariable	x	x	96	95	x	x	94	91
Tree Height	96	96	95	96	95	96	96	95
Yule Model	95	95	95	92	95	96	97	95
birth Rate	96	93	95	91	94	93	94	93
ucldStdev	96	93	93	96	97	97	96	90
frequencies.1	x	x	x	x	96	96	97	96
frequencies.2	x	x	x	x	97	92	96	96
frequencies.3	x	x	x	x	93	96	92	92
frequencies.4	x	x	x	x	96	98	93	90
frequencies.5	x	x	x	x	95	93	97	95
frequencies.6	x	x	x	x	98	94	94	91
frequencies.7	x	x	x	x	94	97	95	95
frequencies.8	x	x	x	x	99	89	91	92
frequencies.9	x	x	x	x	92	93	94	96
frequencies.10	x	x	x	x	97	96	94	98
frequencies.11	x	x	x	x	95	91	93	93
frequencies.12	x	x	x	x	96	92	97	95
frequencies.13	x	x	x	x	98	93	95	96
frequencies.14	x	x	x	x	93	97	92	93
frequencies.15	x	x	x	x	94	96	96	95
frequencies.16	x	x	x	x	95	93	96	91
frequencies.17	x	x	x	x	93	97	89	92
frequencies.18	x	x	x	x	92	92	98	91
frequencies.19	x	x	x	x	96	91	96	94
frequencies.20	x	x	x	x	94	96	95	98

#### Identifiability of gamma shape and proportion of invariable sites

When both gamma rate heterogeneity and invariable sites are in the true model under an exponential prior with lower bound zero (see Table S4), the estimates are slightly diminished due to lack of identifiability as explained before and observed by Bouckaert & Drummond (2017). However, when a lower bound of 0.1 is used as motivated by our discussion, identifiability increases as witnessed by the expected coverage of gamma shape and proportion of invariable sites estimates in Table 2.

**Figure 3:**
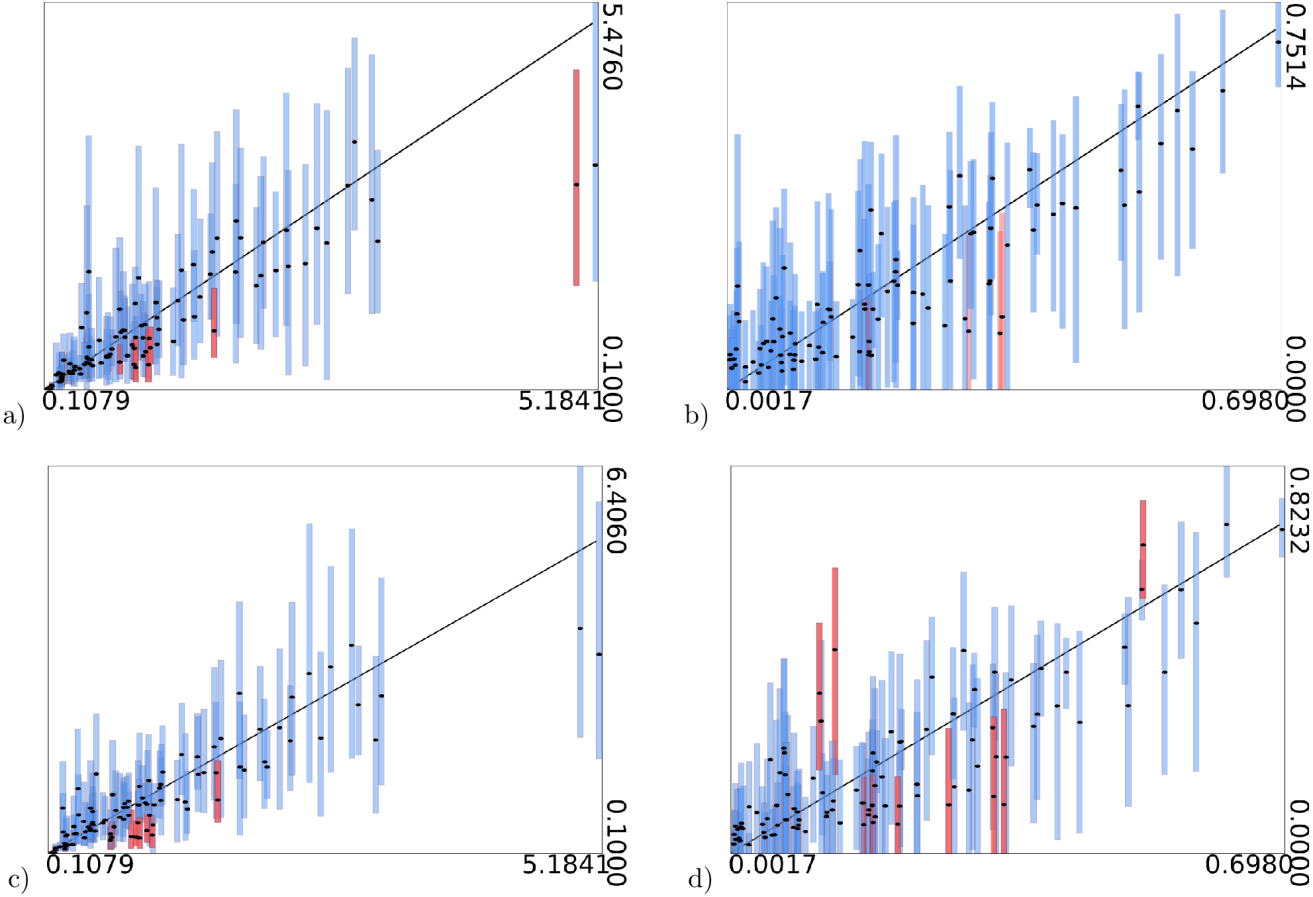
True values of gamma shape parameter (A and C) and proportion of invariable sites (B and D) on *x*-axis vs estimated intervals on *y*-axis. (A) and (B) are when model frequencies are used, and (C) and (D) when frequencies are sampled from a Dirichlet(4,4,…,4) distribution. Black dots represent mean of the estimates. Bars represent 95% HPD intervals, the black diagonal line is where *x*-axis and *y*-axis have equal values. Blue bars contain the true value, red bars miss out on the true value.

Furthermore, as shown in Fig. 3, the estimates of these parameters are informed by the data and not only the prior: when values used to simulate the data increase, their estimates increase as well.

#### Variants on simulation study

The simulation study was repeated with 50 instead of 200 amino acids, providing more uncertainty and thus larger estimates in 95% HPD intervals, but coverage remained good (Table S3). The lower bound for *α* was 0 in these experiments. It was also repeated using a strict clock, and inference done with a strict clock (Table S1) as well as under a relaxed clock (Table S2) with similar results.

#### Frequency operator

**Figure 4:**
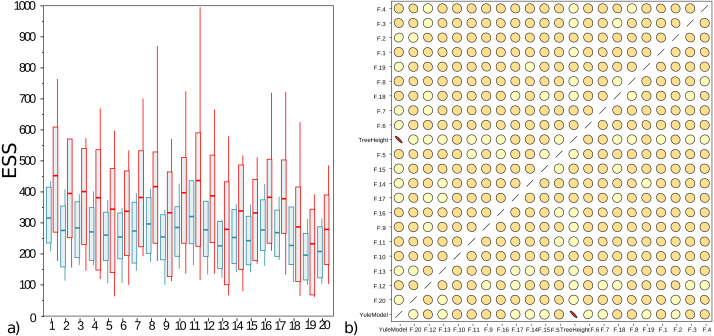
(A) Box plot of effective sample sizes for frequencies for 100 runs when a delta exchange operator is used (blue) and when the adaptive variance multivariate normal is used (red). The AVMN operator is more efficient most of the time. (B) Correlations between estimated frequencies for one of the simulated runs: darker colours and more skewed ovals indicate higher correlations. For contrast, tree height and Yule density are included, which are highly correlated. Frequencies on the other hand show very low correlation. This is typical for the correlation between frequencies that were spot checked. Both plots were generated by Tracer (Rambaut et al., 2018).

The well calibrated study from Table 2 uses the AVMN operator. The simulation experiment was repeated with delta exchange and AVMN operator, both with a weight of 1, giving them equal probability of being selected, and all other operators unaltered. Run times for both experiments were very similar, even though the AVMN operator performs a relatively large amount of work to produce a proposal. The effective sample sizes (ESSs) for 100 runs for the simulation experiment are shown in Fig. 4A, showing the AVMN operator outperforming the delta exchange operator: in all cases the average ESS increases, though the variance of ESSs from MCMC is usually quite high.

## Discussion

Figure 4B shows that correlations between frequencies is very low, so the AVMN operator may not benefit from estimating all covariances. It appears the AVMN operator performs more work than strictly necessary, given that it estimates the complete covariance matrix Σ, even thought (as suggested in Fig. 4B), there is very little correlation between frequencies. However, since the AVMN operator does not seem to require noticeable overhead (using a profiler does not show any load from the operator and run times were very similar to using a delta exchange operator), further optimisation will be hard to justify.

### Site models matter

To determine the effectiveness of the OBAMA model, we investigated an amino acid alignment, M200 from TreeBase^1^ 1TreeBASE Study URI: http://purl.org/phylo/treebase/phylows/study/TB2:S795
(Simmons et al., 2002). This alignment for 20 flowering plant taxa is 466 characters long. ProtTest (Darriba et al., 2011) (run with settings -all-matrices -all-distributions -F -threads 2) suggests JTT+G+I+F fits best (where +G stands for using gamma rate heterogeneity, +I using invariable sites, and +F using observed frequencies), followed by WAG+G+I+F and LG+G+I+F as third runner up, both based on the BIC and the Ln criteria.

**Figure 5:**
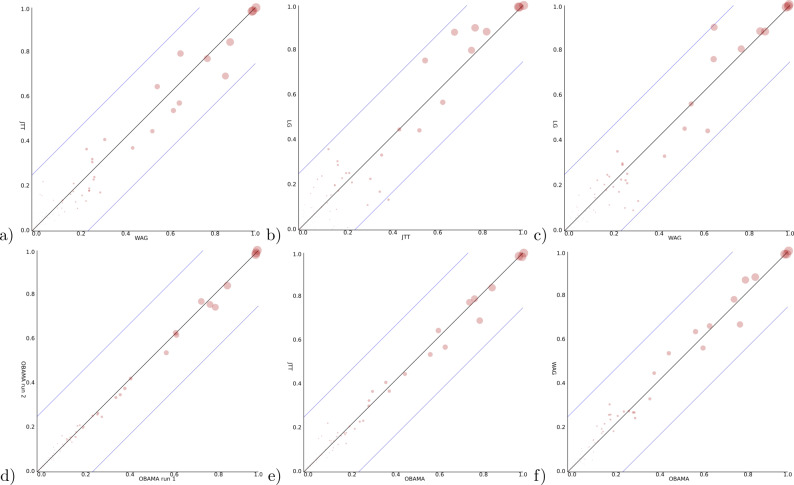
Clade support of an analysis for all clades in a posterior tree set vs. support for the same clade in another analysis. Diagonal lines indicate area where at most 25% difference in clade support is observed. (A) WAG (*y*-axis) vs JTT (*x*-axis) has max clade support difference of 17.3% (B) JTT vs. LG (max difference of 27.1%) (C) WAG vs. LG (23.4%) (D) OBAMA vs. OBAMA separate runs, illustrating (low) variance between runs (6.7%) (E) OBAMA vs. JTT (11.0%) (F) OBAMA vs. WAG (11.8%).

For a Bayesian analysis, a relaxed log normal clock (Drummond et al., 2006) can be rejected based on a coefficient of variation being distributed with mean less than 0.1 skewed towards zero. So, we use a strict clock in the remainder, a Yule prior with birth rate estimated and vary the site model using the three models suggested by ProtTest. All runs were verified to have ESSs exceeding 200 and four independent runs converging to the same distribution. Data files used can be found at https://github.com/rbouckaert/obama/releases/tag/data. When we compare the posterior clade support for these models (Figs. 5A–5C), we see considerable differences. The largest clade support difference between WAG and JTT is 17.3%, while between JTT and LG as well as WAG and LG these exceed 20%. As comparison, two independent runs of OBAMA only show a difference of just 6.7% in clade support. This indicates the three different models sample from different distributions and the choice of substitution model matters for the analysis.

With the OBAMA model, about 84% of the time JTT is sampled and 16% WAG while the other substitution models have no recorded posterior support. Gamma rate heterogeneity, proportion of invariable sites and estimated frequencies were supported 100% of the time. Comparing the three individual substitution models with OBAMA (Figs. 5E and 5F) shows that JTT and WAG have differences in clade support of 11.0% and 11.8% respectively, which shows that model averaging has an effect on the posterior distribution, and matters as well.

## Conclusions

We implemented a new user friendly site model for performing Bayesian phylogenetic analyses of amino acid sequences. There are no tuning parameters, and since the site model is usually of no interest to the question at hand, it takes the tedium out of having to specify the details of a site model by averaging over a range of models. By careful choice of a prior, it allows identifiability of gamma shape and proportion of invariable sites. We observed that the site model matters, and has a substantial effect on posterior supports of clades in a tree. Likewise, Bayesian averaging over models affects clade support as well, and arguably represents uncertainty of the site model details more appropriately.

**Figure 6:**
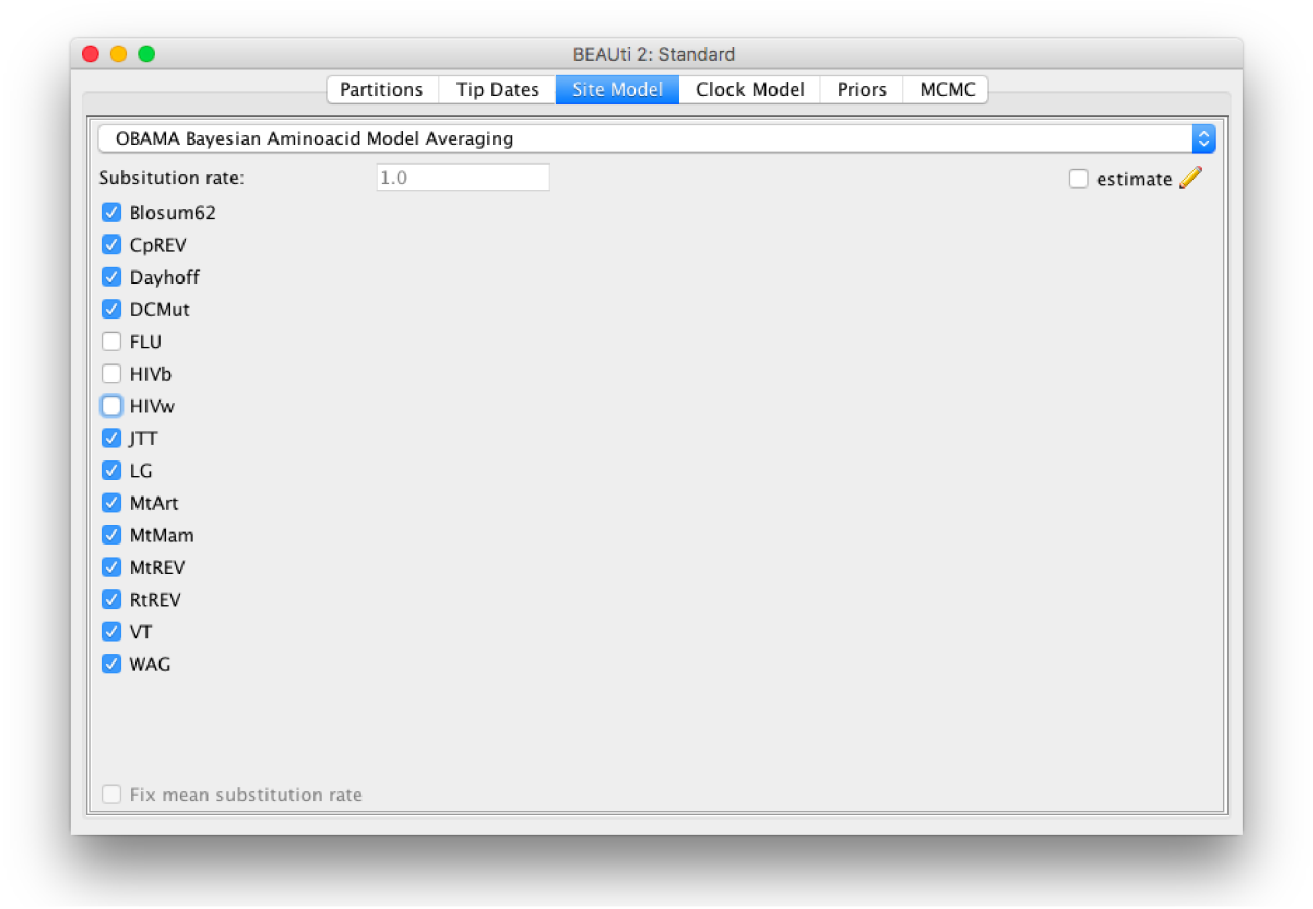
Graphical user interface for setting up an OBAMA analysis. There is a choice of 15 substitution models that can be included or excluded from the analysis. When there are multiple partitions, relative substitution rates among these partitions can be estimated.

The method is implemented in the OBAMA package in BEAST 2, and has a graphical user interface (Fig. 6). It is open source licensed under LGPL and allows joint tree inference under a wide range of models, which allows combining the analysis with phylogeographical information, data from the fossil record, species delimitation, etc. The range of empirical substitution models can be easily extended by simply adding an appropriate reference to the model in the BEAST XML when more empirical models become available.

There are several other ways the model can be extended. For example, by using a mixture of empirical rate matrices by replacing the model indicator (which effectively puts 100% support on a single matrix) with a weight vector and create a new rate matrix as the weighted sum of empirical matrices. There is also potential to extend in the direction of grouping sites into categories, each having their own combination of OBAMA model, similar to the SubstBMA model for nucleotides (Wu, Suchard & Drummond, 2013), which has shown a remarkable increase in fit over single model use for a partition. Another avenue, though one that requires a bit more thought regarding priors and MCMC proposals, is to combine OBAMA with other frequency models like the free rate model (Soubrier et al., 2012) and include mixture models like LG4X (Le, Dang & Gascuel, 2012).

## Supplemental Information

10.7717/peerj.9460/supp-1 Supplemental Information 1 Supplementary tables

## Additional Information and Declarations

### Data Availability

The following information was supplied regarding data availability: All code and data are available at GitHub: https://github.com/rbouckaert/obama/.
