# Supplementary material for: OBAMA: OBAMA for Bayesian amino-acid model averaging
Source: PeerJ. 2020 Aug 4;8:e9460. doi: 10.7717/peerj.9460 (PMC7413081; doi:10.7717/peerj.9460)
Supplement: Supplemental Information 1 [file peerj-08-9460-s001.pdf]

1 SUPPLEMENTARY TABLES

True model:	-	+G	+I	+G+I	+F	+G+F	+I+F	+G+I+F
Model Indicator	100	98	99	98	99	100	100	98
has Invariable Sites	100	99	100	100	100	98	100	100
has Gamma Rates	100	96	98	96	100	97	99	100
use External Freqs	100	100	100	100	100	100	98	98
gamma Shape	x	100	x	93	x	97	x	91
Proportion Invariable	x	x	95	94	x	x	94	94
Tree Height	96	93	91	94	100	94	98	97
Yule Model	92	88	93	95	98	95	98	98
birth Rate	93	92	92	92	93	92	92	93
frequencies.1	x	x	x	x	94	94	91	91
frequencies.2	x	x	x	x	93	96	97	95
frequencies.3	x	x	x	x	92	90	91	90
frequencies.4	x	x	x	x	94	94	94	92
frequencies.5	x	x	x	x	93	97	94	93
frequencies.6	x	x	x	x	93	90	94	95
frequencies.7	x	x	x	x	93	97	95	97
frequencies.8	x	x	x	x	94	98	93	96
frequencies.9	x	x	x	x	93	98	95	90
frequencies.10	x	x	x	x	91	92	92	92
frequencies.11	x	x	x	x	98	91	93	92
frequencies.12	x	x	x	x	91	97	94	95
frequencies.13	x	x	x	x	94	92	93	93
frequencies.14	x	x	x	x	95	94	90	94
frequencies.15	x	x	x	x	97	96	96	96
frequencies.16	x	x	x	x	94	94	95	91
frequencies.17	x	x	x	x	99	99	94	94
frequencies.18	x	x	x	x	97	93	95	93
frequencies.19	x	x	x	x	94	95	89	94
frequencies.20	x	x	x	x	96	92	93	96

Table S1. As Table 2 but for sequences with length 50 instead of 200, strict clock instead of relaxed clock to simulate data as well as doing inference.

True model:	-	+G	+I	+G+I	+F	+G+F	+I+F	+G+I+F
Model Indicator	99	100	97	99	100	100	99	99
has Invariable Sites	100	98	100	100	100	98	100	100
has Gamma Rates	100	96	98	98	100	99	99	100
use External Freqs	100	99	100	99	97	100	100	99
gamma Shape	x	96	x	91	x	94	x	93
Proportion Invariable	x	x	95	96	x	x	97	98
Tree Height	97	90	94	93	97	92	91	94
Yule Model	99	91	94	92	97	96	98	95
birth Rate	94	90	92	93	93	94	91	93
ucldStdev	96	98	97	96	96	97	95	97
frequencies.1	x	x	x	x	98	94	93	92
frequencies.2	x	x	x	x	95	95	94	88
frequencies.3	x	x	x	x	91	94	96	96
frequencies.4	x	x	x	x	95	96	89	90
frequencies.5	x	x	x	x	96	95	91	94
frequencies.6	x	x	x	x	97	94	98	95
frequencies.7	x	x	x	x	96	94	94	93
frequencies.8	x	x	x	x	98	95	96	99
frequencies.9	x	x	x	x	90	94	96	91
frequencies.10	x	x	x	x	95	96	93	93
frequencies.11	x	x	x	x	90	95	93	88
frequencies.12	x	x	x	x	94	90	92	95
frequencies.13	x	x	x	x	92	95	93	90
frequencies.14	x	x	x	x	87	91	94	89
frequencies.15	x	x	x	x	95	92	87	92
frequencies.16	x	x	x	x	96	91	96	96
frequencies.17	x	x	x	x	93	95	96	93
frequencies.18	x	x	x	x	93	95	94	88
frequencies.19	x	x	x	x	93	91	94	92
frequencies.20	x	x	x	x	92	94	92	97

Table S2. As Table 2 but for sequences with length 50 instead of 200, strict clock instead of relaxed clock to simulate data, but relaxed clock to infer model.

True model:	-	+G	+I	+G+I	+F	+G+F	+I+F	+G+I+F
Model Indicator	100	100	100	100	100	100	100	100
has Invariable Sites	100	99	100	100	100	98	100	100
has Gamma Rates	100	98	100	99	100	98	99	99
use External Freqs	100	100	100	99	100	100	99	100
gamma Shape	x	96	x	91	x	94	x	98
Proportion Invariable	x	x	91	96	x	x	97	94
Tree Height	97	95	93	97	97	98	96	97
Yule Model	96	93	94	95	96	100	98	94
birth Rate	92	93	91	92	92	93	93	92
ucldStdev	96	98	97	97	96	97	97	96
frequencies.1	x	x	x	x	92	95	97	91
frequencies.2	x	x	x	x	92	95	91	94
frequencies.3	x	x	x	x	95	92	94	96
frequencies.4	x	x	x	x	92	94	91	93
frequencies.5	x	x	x	x	94	95	95	95
frequencies.6	x	x	x	x	93	94	93	91
frequencies.7	x	x	x	x	95	95	95	95
frequencies.8	x	x	x	x	92	96	96	93
frequencies.9	x	x	x	x	92	97	96	95
frequencies.10	x	x	x	x	97	92	93	94
frequencies.11	x	x	x	x	95	94	94	89
frequencies.12	x	x	x	x	92	92	93	93
frequencies.13	x	x	x	x	88	93	96	94
frequencies.14	x	x	x	x	95	94	93	95
frequencies.15	x	x	x	x	92	96	95	96
frequencies.16	x	x	x	x	96	91	98	91
frequencies.17	x	x	x	x	92	92	92	93
frequencies.18	x	x	x	x	90	92	94	96
frequencies.19	x	x	x	x	94	91	94	93
frequencies.20	x	x	x	x	95	92	93	89

Table S3. As Table 2 but for sequences with length 50 instead of 200.

True model:	-	+G	+I	+G+I	+F	+G+F	+I+F	+G+I+F
model indicator	100	99	100	100	100	100	100	100
has invariable sites	100	98	98	100	100	97	100	100
has gamma rates	100	98	99	97	100	99	99	99
use external freqs	100	100	100	100	100	100	100	100
gamma shape	x	93	x	91	x	92	x	95
proportion invariable	x	x	93	89	x	x	94	89
tree height	94	91	90	96	91	92	94	94
Yule model	93	90	93	97	93	94	96	95
birth rate	92	93	92	92	93	92	92	92
UCLD stdev	96	96	97	96	95	94	97	97
frequencies.1	x	x	x	x	96	95	93	94
frequencies.2	x	x	x	x	95	96	97	94
frequencies.3	x	x	x	x	92	92	91	92
frequencies.4	x	x	x	x	95	96	92	96
frequencies.5	x	x	x	x	96	97	94	94
frequencies.6	x	x	x	x	94	95	93	92
frequencies.7	x	x	x	x	94	95	98	94
frequencies.8	x	x	x	x	96	95	96	95
frequencies.9	x	x	x	x	95	95	95	89
frequencies.10	x	x	x	x	96	95	97	97
frequencies.11	x	x	x	x	95	89	97	94
frequencies.12	x	x	x	x	96	96	97	92
frequencies.13	x	x	x	x	92	94	95	94
frequencies.14	x	x	x	x	95	94	97	93
frequencies.15	x	x	x	x	95	95	93	98
frequencies.16	x	x	x	x	93	98	96	92
frequencies.17	x	x	x	x	93	95	96	95
frequencies.18	x	x	x	x	95	96	94	93
frequencies.19	x	x	x	x	95	93	95	95
frequencies.20	x	x	x	x	98	90	94	98

Table S4. As Table 2 but prior on gamma shape not truncated at 0.1.

True model:	-	+G	+I	+G+I	+F	+G+F	+I+F	+G+I+F
Model Indicator	100	100	99	100	98	99	100	99
has Invariable Sites	100	99	100	100	100	99	100	100
has Gamma Rates	100	97	100	98	100	98	96	97
use External Freqs	100	99	98	100	99	100	100	99
gamma Shape	x	98	x	91	x	97	x	93
Proportion Invariable	x	x	93	92	x	x	95	93
Tree Height	96	96	97	94	94	94	98	99
Yule Model	90	95	92	91	94	95	99	96
birth Rate	97	96	96	96	95	96	96	95
ucldStdev	95	94	99	94	93	95	92	97
frequencies.1	x	x	x	x	96	96	93	98
frequencies.2	x	x	x	x	91	96	99	99
frequencies.3	x	x	x	x	95	88	92	94
frequencies.4	x	x	x	x	95	97	93	96
frequencies.5	x	x	x	x	96	96	96	94
frequencies.6	x	x	x	x	93	94	93	96
frequencies.7	x	x	x	x	92	95	93	92
frequencies.8	x	x	x	x	96	95	91	94
frequencies.9	x	x	x	x	95	97	97	94
frequencies.10	x	x	x	x	95	96	91	92
frequencies.11	x	x	x	x	90	94	92	92
frequencies.12	x	x	x	x	94	93	90	93
frequencies.13	x	x	x	x	98	92	96	96
frequencies.14	x	x	x	x	96	92	96	93
frequencies.15	x	x	x	x	92	95	96	95
frequencies.16	x	x	x	x	96	93	94	95
frequencies.17	x	x	x	x	98	96	95	93
frequencies.18	x	x	x	x	95	95	94	94
frequencies.19	x	x	x	x	97	94	94	95
frequencies.20	x	x	x	x	96	97	96	92

Table S5. As Table 2 but with sequences of length 50.
